# Uncovering Structure—Rating Associations in Animated Film Character Networks

**DOI:** 10.3390/e27090914

**Published:** 2025-08-29

**Authors:** Jue Zeng, Yiwen Tang, Xueming Liu

**Affiliations:** The MOE Engineering Research Center of Autonomous Intelligent Unmanned Systems, State Key Laboratory of Digital Manufacturing Equipment and Technology, School of Artificial Intelligence and Automation, Huazhong University of Science and Technology, Luoyu Road, Wuhan 430074, China; zengjuehuster@outlook.com (J.Z.); yiwent@126.com (Y.T.)

**Keywords:** complex network, character network, film ratings, semantic distances

## Abstract

The narrative structure of animated films plays a critical role in shaping audience perception, yet quantitative investigations into how character interaction networks influence film ratings remain limited. To address this gap, we apply complex network theory to analyze 82 animated films, extracting character networks from narrative interactions and examining key topological features—including centrality heterogeneity, protagonist relative centrality, network density, clustering coefficient, average shortest path length, and semantic diversity of relationships. Our findings demonstrate that higher-rated films are characterized by greater disparities in character centrality, lower network density and efficiency, longer average shortest path lengths, and richer semantic diversity. These structural patterns suggest that loosely connected yet hierarchically organized character networks enhance narrative complexity and audience engagement. The proposed framework offers a quantitative, data-driven approach to narrative design and provides a theoretical foundation for analyzing storytelling structures across diverse media, including novels, television series, and comics.

## 1. Introduction

**Figure 1:**
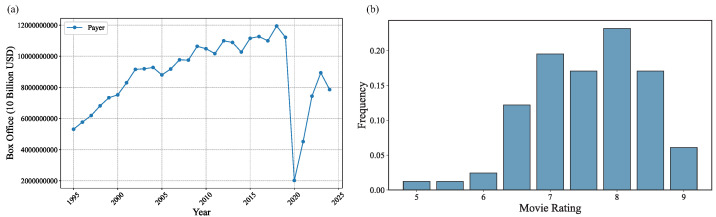
(**a**) Global box office revenues of animated films [6]. (**b**) Rating frequency of 82 animated films on Douban [7].

Animated films occupy a significant position in the global film industry due to their unique artistic characteristics and broad audience appeal. As illustrated in Figure 1, the global box office revenues of animated films increased steadily from 1995 to 2019, highlighting the extensive market appeal of this genre. In 2020, the global box office was significantly impacted by the COVID-19 pandemic. Consequently, the global film industry is currently in a crucial recovery phase. High-quality films not only stimulate the revival of the film industry but also contribute to broader economic recovery. In addition, animated films are particularly well-suited for this study, as their unique visual nature allows for a more flexible narrative approach, free from the constraints of real-world genres and structures, making them a valuable and representative sample for analysis.

High-quality films often exhibit distinct network characteristics that enhance narrative complexity and audience engagement. The interactions between characters in films have certain notable features. From the perspective of network analysis, character networks in high-quality films possess clear advantages and unique structures. Analyzing these character networks helps identify key factors determining film quality, thus providing effective guidance for creating high-quality films. Moreover, for high-budget films, predicting audience reception is crucial to optimizing narrative development, character progression, and investment decisions [1].

However, existing approaches to audience prediction and narrative guidance primarily rely on the characteristics of actors, directors, movie genres [2], and film reviews [3,4]. These approaches require detailed information typically available only after film production or during advanced planning stages, thus limiting their effectiveness in guiding early narrative design.

Character interactions play a foundational role in film narration by driving plot development, revealing character traits, deepening themes, and fostering emotional resonance with audiences [5]. Because of this centrality, systematically examining their structure is vital to understanding film quality and audience reception. Traditional qualitative analyses provide valuable insights but often lack the resolution to capture complex relationship patterns. Network analysis fills this gap by modeling characters as nodes and their interactions, such as dialogues, conflicts, and alliances, as edges in a graph. Quantifying network topology reveals structural signatures, including the small-world properties frequently observed in critically acclaimed films, which remain underexplored in current narrative theory. Linking these signatures to ratings and box office data offers practical guidance for crafting narratively engaging and commercially successful stories.

Network analysis provides an objective framework for evaluating films by converting intricate character relationships into quantifiable metrics. Unlike traditional qualitative approaches, this method precisely identifies interaction patterns and their roles within the narrative structure. Quantitative examination of character networks systematically reveals latent alliances, central conflict points. and critical turning events [8]. These hidden patterns, often overlooked during scriptwriting, nonetheless shape audience experience and overall film quality. Moreover, network metrics can predict audience responses: a character network with many indirect connections, for example, usually indicates a more complex narrative and higher audience engagement. By modeling characters as nodes and their dialogues, emotional conflicts, or alliances as edges, the resulting network offers a visual, measurable representation of narrative dynamics.

In contemporary scientific research, complex-network theory has emerged as a robust analytical framework, applied across sociology, biology, physics, and information science. At its core, the theory represents interactions among system components as networks of nodes and edges. This representation not only reveals the system’s intrinsic structure but also uncovers its dynamic behavior and functional characteristics through quantitative analysis. In film studies, for instance, degree quantifies character activity and interaction frequency, while betweenness and closeness centralities pinpoint pivotal characters and their narrative influence. The clustering coefficient captures tightly knit subgroups, and network density gauges the overall intensity of interactions. Crucially, complex-network analysis is highly adaptable: it works for genres ranging from linear plots to multi-threaded stories, enabling systematic within-film exploration and cross-film comparison. By exposing internal narrative structures and the common signatures of successful films, it offers actionable insights for scholars and the industry alike.

Several studies have investigated film character networks. For example, Lingfei Zhang et al. [9] conducted a preliminary exploration of factors influencing the popularity of films and television series, but their sample contained only three TV series, limiting the generalizability of their findings. Community structures in screen narratives have also been examined with social-network methods [10]. Beyond traditional screen media, complex-network analysis has been applied to the Marvel Universe, revealing topological properties of character networks extracted from comics and related franchises [11,12]. More recent work extracts networks directly from audiovisual content, including story-based models built on dialogue co-occurrence [13] and multimodal approaches that fuse textual, visual, and acoustic cues [14]. This study leverages character-network analysis to give filmmakers quantitative insight into relationship structures, streamlining iterative screenwriting and increasing the likelihood of a film’s success. Grounded in complex-network theory, we examine how topological features of character networks correlate with audience ratings, thereby establishing a unified analytical framework applicable across diverse genres. Using the dataset of animated features, we show that specific network metrics predict reception and unveil narrative patterns that underpin cinematic impact.

## 2. Materials and Methods

### 2.1. Data Collection and Sources

**Figure 2:**
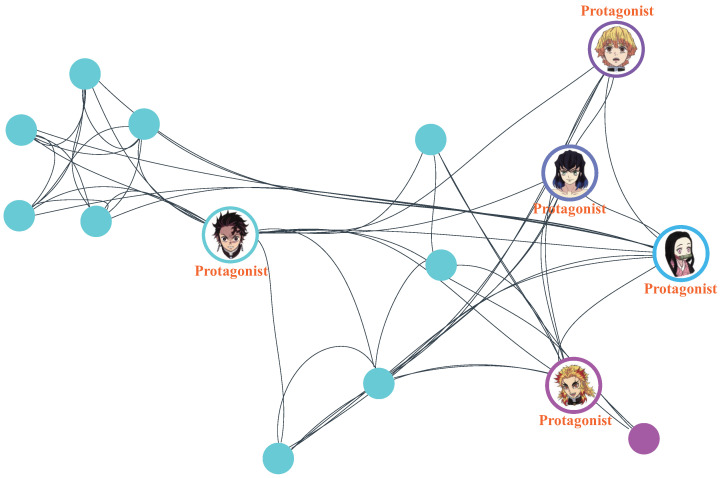
Character networks of *Demon Slayer: Kimetsu no Yaiba—The Movie: Mugen Train*.

Focusing on animated films, we obtained their Douban ratings and retrieved their corresponding character networks from the encyclopedic atlas section of Baidu Baike. The character-network data for animated films were acquired by scraping the names and ratings of animated films from Douban and subsequently retrieving the corresponding character networks from Baidu Baike based on these film names. A total of 82 character networks were collected and analyzed alongside their Douban ratings; the frequency distribution of these ratings is shown in Figure 1b. Douban is the largest film sharing and review community in China [15]. Unlike Rotten Tomatoes, Douban employs a model similar to IMDb, allowing the general public to rate films on a scale from 0 to 10. After processing these data, we derived 82 animated film character networks, each labeled with the protagonists and relational edges. As shown in Figure 2, we provide an example of such a network (with character relationships omitted) to illustrate the structural representation.

### 2.2. Metrics for Character Influence Differential

#### 2.2.1. Character Influence Reflected Through Centrality

Centrality measures are fundamental tools in network analysis, quantifying the importance of nodes within a network. In the context of movie character networks, these metrics reveal which characters play pivotal roles in driving the narrative, influencing other characters, and shaping the overall story structure. The centrality measures employed in this study include degree centrality, betweenness centrality, and harmonic centrality.

Degree centrality, as defined in [16], measures the number of direct connections a node has with other nodes. A character with high degree centrality typically engages in numerous interactions, highlighting their central role within the narrative. For example, in a film, a protagonist who frequently interacts with other characters demonstrates high degree centrality, underscoring their significance in the storyline. However, while degree centrality effectively reflects a node’s local influence, it does not fully capture its global influence within the entire network [17].

Brian Mullen et al. [17] identified both betweenness centrality and degree centrality as important predictors of leadership. John Scott [18] further noted that betweenness centrality captures the extent to which an agent functions as a “broker” or “gatekeeper” and thus has the potential to influence others. Specifically, betweenness centrality measures how often a character lies on the shortest paths between pairs of other characters, thereby acting as a bridge that links different subgroups within the network. Characters with high betweenness centrality frequently mediate conflicts, facilitate communication, and control information flow. For example, in a film with multiple plotlines, a character who regularly interacts with figures from distinct subplots would have high betweenness centrality, underscoring that character’s importance in maintaining narrative coherence.

Murray A. Beauchamp [19] improved the concept of closeness centrality, a metric designed to assess a node’s ability to efficiently disseminate information across a network. However, closeness centrality can produce unstable or even meaningless results in certain network structures, particularly in those that are disconnected or sparse, due to the influence of extreme nodes. To address this issue, Yannick Rochat [20] proposed harmonic centrality, which effectively identifies central nodes and provides valuable insights into their local influence within such networks [21].

The coefficient of variation (CV) is the ratio of the standard deviation to the mean. Because it normalizes variability by the mean, the CV enables meaningful comparisons across datasets that differ in scale or units. Within character networks, the CV can gauge how evenly influence is distributed: computing the CV of centrality metrics (such as degree or betweenness centrality) shows whether influence is concentrated in a few protagonists or spread more uniformly across the cast. Formally,

(1) $$\mathrm{CV}=\frac{\sigma }{\mu }$$

where σ is the standard deviation and μ is the mean.

Films with high CV values for degree centrality are typically built around one or more protagonists or a very small core group whose connections dominate the network. Most other characters link directly to these central figures, so the hierarchy is sharp and viewers naturally follow the main protagonists’ arcs.

Films with low CV values for betweenness centrality still revolve around clusters centered on the protagonists, but the differences between leading and supporting roles in their bridging positions are modest. Betweenness values are similar across the cast, so no single character monopolizes the task of linking subgroups. Influence remains led by the protagonists, yet the gap between primary and secondary roles is small.

To further validate the findings derived from CV analysis, we examined the coefficient of variation of character centrality across a selected subset of animated films. This comparison revealed clear patterns in narrative structure and in how influence is distributed among characters.

**Figure 3:**
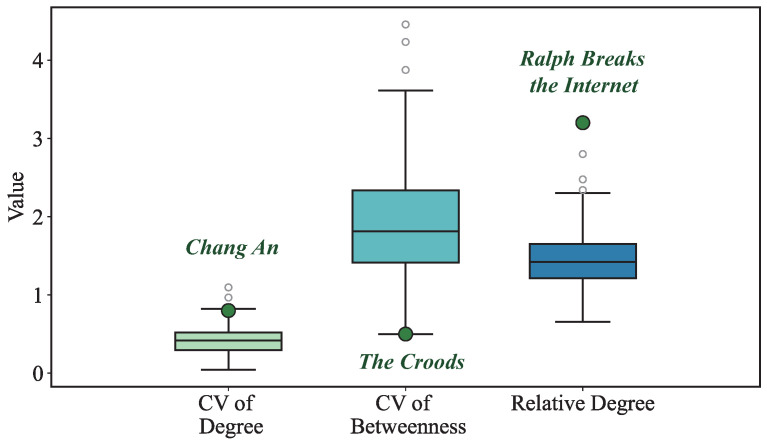
Distribution of global features and movie-specific values.

For example, in *Chang An*, the high CV for degree centrality (Figure 3) indicated a clear protagonist-driven narrative, which aligned with its traditional storytelling style. In contrast, *The Croods* exhibited a lower CV for betweenness centrality (Figure 3), reflecting its more complex, multi-character narrative structure. This comparative analysis highlighted the diversity in narrative approaches and provided a quantitative basis for understanding how different films achieve their storytelling goals.

A higher coefficient of variation in character centrality indicates a pronounced disparity between leading and supporting roles, resulting in a well-defined hierarchical structure. This hierarchy foregrounds the narrative core and thematic focus while providing a clear framework for character development and plot progression. Because films typically revolve around one or several principal characters, the character network should be designed to emphasize their central position in the topology, ensuring an orderly allocation of narrative elements and a clearly maintained story focus.

#### 2.2.2. Relative Centrality of Protagonists

In film narratives, protagonists are generally expected to occupy central positions within the character network, driving the story forward and influencing others. Their centrality can be described with three measures. Degree centrality indicates the number of direct ties a protagonist holds. Betweenness centrality shows how often the protagonist lies on shortest paths that link separate subgroups. Harmonic centrality reflects how efficiently the protagonist can reach every other character, thereby supporting overall network coherence.

To evaluate these properties, we calculated degree, betweenness, and harmonic centrality for each protagonist in our sample of 82 animated films. Each value was then expressed as a ratio to the mean centrality of all characters in the same film. In Ralph Breaks the Internet, for example, the protagonist exhibits high degree centrality, signaling frequent direct interactions with many characters and an active presence across multiple storylines. The same protagonist also shows high betweenness centrality, underlining a bridging role that mediates conflicts and sustains narrative coherence.

By quantifying protagonist centrality with metrics such as degree and betweenness centrality, we gain deeper insight into the role of protagonists in driving the narrative and influencing other characters. Our analysis highlights the importance of protagonist centrality in enhancing audience engagement and film ratings, providing valuable guidance for film analysis and creation.

### 2.3. Metrics for Strength of Characters’ Relationships

In film narratives, relationships among characters are central to both narrative structure and audience engagement. The closeness of these ties, captured by network density and the average clustering coefficient, can strongly influence how viewers perceive and rate a movie. Understanding these interaction patterns offers filmmakers and analysts valuable insights into the ways character dynamics drive plot development and shape audience reception.

#### 2.3.1. Characters’ Relationships and Network Density

Network density is a fundamental metric in network analysis, defined as the ratio of the actual number of edges (interactions) present in a network to the maximum possible number of edges within that network. It quantifies the overall connectivity of the network, reflecting the frequency and intensity of direct interactions between characters. A high network density indicates a tightly knit network where characters frequently interact with each other, while low density suggests a more sparse network with fewer direct connections. Mathematically, network density (*D*) is calculated as

(2) $$D=\frac{2m}{n(n-1)}$$

where *n* represents the number of nodes and *m* denotes the number of edges in network [22].

#### 2.3.2. Characters’ Relationships and Clustering Coefficient

The local clustering coefficient measures the extent to which a node’s neighbors are interconnected. It reflects the degree to which characters form tightly knit groups or clusters within the network. The average clustering coefficient provides an overall measure of how interconnected the network is at the local level. A high clustering coefficient indicates that characters tend to form dense subgroups, while a low coefficient suggests a more fragmented network structure. Mathematically, the local clustering coefficient ($C_{i}$) for a node *i* is calculated as

(3) $$C_{i}=\frac{2E_{i}}{k_{i}\left(k_{i}-1\right)}$$

where $E_{i}$ represents the actual number of edges between the neighbors of node *i* and $k_{i}$ denotes the degree of node *i*.

Intense direct interactions, reflected by high network density and high clustering coefficients, can influence audience ratings in contrasting ways. On one hand, frequent character interactions create a rich and dynamic narrative environment that broadens thematic complexity and deepens the storyline, thereby attracting viewers with multiple points of interest. On the other hand, excessive interconnectedness may leave little room for suspense or narrative surprise, potentially leading to a crowded and less focused plot.

### 2.4. Metrics for Information Transmission Between Characters

In film narratives, the efficiency of information transmission among characters is closely linked to audience reception. When information flows too efficiently and directly, interactions become frequent and transparent, reducing opportunities for foreshadowing and suspense and weakening dramatic tension. By contrast, a moderate degree of information blockage, reflected in longer average shortest path lengths and lower network efficiency, can conceal key details, delay plot revelations, and build suspense while gradually advancing the story. This study employs average shortest path length (ASPL) and network efficiency to quantify the cost and effectiveness of information transmission in character networks and examines how these metrics relate to film ratings.

#### 2.4.1. Characters’ Information Transmission and Average Shortest Path Length

The ASPL measures the average number of steps along the shortest paths for all possible pairs of nodes in a network. In the context of film character networks, ASPL reflects the efficiency of information exchange between characters. A shorter path length indicates that information can travel more quickly and efficiently between characters, while a longer path length suggests delays or potential bottlenecks in information flow. Mathematically, the ASPL is defined as

(4) $$L=\frac{1}{N(N-1)}\sum_{i\neq j\in G}d_{ij}$$

where $d_{ij}$ denotes the shortest path from node *i* to node *j*, and *N* is the total number of nodes in the network. In simpler terms, ASPL provides a way to measure how well-connected the characters are, and how easily they can exchange information.

#### 2.4.2. Characters’ Information Transmission and Network Efficiency

Network efficiency, introduced by Vito Latora and Massimo Marchiori [23], measures how efficiently information is exchanged within a network. Unlike ASPL, which focuses on the shortest paths, network efficiency considers the overall connectivity and redundancy of the network. It is defined as the average inverse shortest path length between all pairs of nodes, normalized by the number of nodes. Mathematically, network efficiency (*E*) is defined as

(5) $$E\left(G\right)=\frac{1}{N(N-1)}\sum_{i\neq j\in G}\frac{1}{d_{ij}}$$

where *G* is an undirected graph, *N* is the number of nodes in network *G*, and $d_{ij}$ represents the shortest path distance from node *i* to node *j*.

The efficiency of information transmission between characters, as measured by ASPL and network efficiency, significantly impacts movie ratings. By balancing suspense and narrative coherence, filmmakers can create compelling stories that engage viewers and maintain their interest. When applied to film narratives, network efficiency, alongside ASPL, reflects the flow of information between characters. This interplay between characters’ relationships and the narrative structure can significantly influence audience engagement and movie ratings.

### 2.5. Diversity and Richness of Characters’ Relationships

The complexity and diversity of relationships between characters are essential elements in shaping a compelling film narrative. Rich and varied character relationships can stimulate conflicts, drive plot development, and provide deeper character portrayals. In this paper, we analyze the impact of the richness of relationships on movie ratings by examining the correlation between animation movie ratings and two key metrics: the number of edge types and the semantic distances of character relationships in semantic space. These metrics help quantify the diversity and complexity of character interactions, providing insights into how these factors influence audience engagement and overall film reception. In character networks, different types of relationships (e.g., friendship, rivalry, romantic interest) can be represented as different edge types. The number of edge types reflects the diversity of relationships within the network. A higher number of edge types indicates a more complex and multifaceted narrative, where characters engage in various types of interactions. This diversity can enhance the depth of character portrayals and create a richer narrative tapestry.

Semantic similarity assessment is a classic task in natural language processing (NLP) that measures the conceptual distance between words or phrases. In the context of film narratives, semantic distances can quantify the diversity of character relationships by analyzing the language used to describe these relationships. By employing pre-trained NLP models, we can assess the semantic distances between different character relationships, providing a quantitative measure of their diversity.

To ensure the reliability of our semantic distance assessments, we employ a variety of pre-trained NLP models, including [chinese-roberta-wwm-ext [24,25], ernie-1.0 [26], and gte-multilingual-base [27]]. These models provide robust and accurate measures of semantic similarity, allowing us to quantify the diversity of character relationships with high precision.

## 3. Results

### 3.1. Analysis of the Basic Topological Features of Character Networks in Animated Films

As shown in Table 1, the number of nodes in the social networks of animated films is relatively small, which aligns with real-world constraints. Due to the time limitations of films, the scale of character networks is inherently smaller compared to other mediums, such as novels or comics. Similar to findings in comics by Kashin Sugishita and Naoki Masuda [28], only four films in our study exhibited a positive-degree assortativity coefficient. This contrasts with the typical characteristics of social networks [29] but has been observed in ego networks [30].

### 3.2. Analysis of Differences in Network Node Centrality Measures

**Figure 4:**
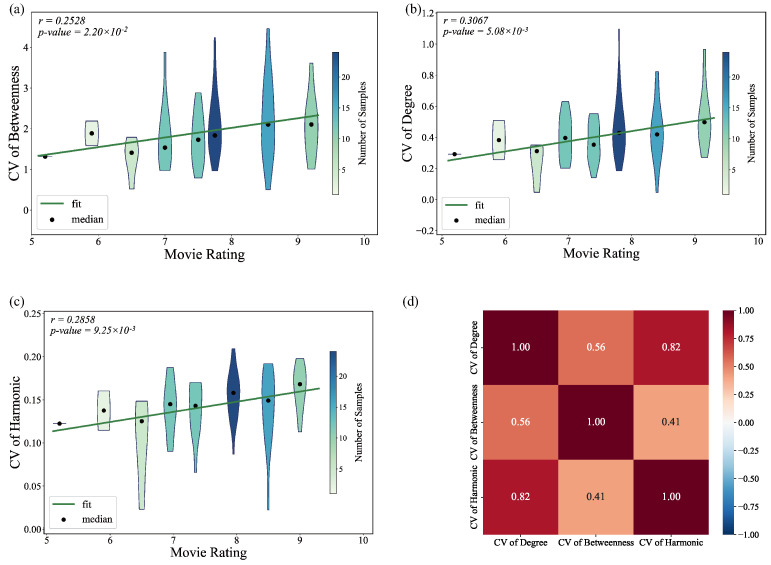
Violin plots showing the relationship between movie ratings and CVs of (**a**) betweenness, (**b**) degree, and (**c**) harmonic. (**d**) Correlation heatmap of CV for different centrality.

Figure 4 illustrates that the CV for degree centrality, harmonic centrality, and betweenness centrality in character networks is positively correlated with film ratings. This suggests that higher variability in these centrality measures is associated with better-rated films. However, no significant correlation was observed between film ratings and the CV of closeness centrality. This lack of correlation is likely due to the very low variability of this measure, as the standard deviation of the CV of closeness centrality across different films is only 0.031.

Additionally, Figure 4d shows that, although harmonic centrality and betweenness centrality are both related to information flow within the network, the correlation between their CV coefficients is relatively low.

**Figure 5:**
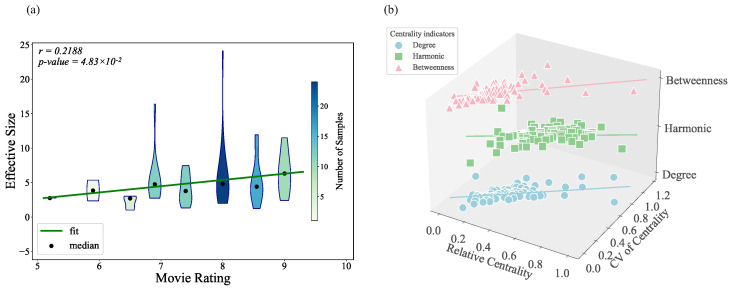
(**a**) The violin plot illustrates the relationship between the effective size of the movie character network and movie ratings. (**b**) The scatter plot shows the CV of network centrality metrics alongside the magnitude of the protagonist’s relative centrality.

To further investigate the source of the centrality differences, the relationship between the centrality of the main characters relative to the overall mean and the CV of centrality is analyzed, as shown in Table 2 and Figure 5b. This analysis reveals that the centrality differences primarily arise from the disparity between main and supporting characters. Furthermore, when examining the relationship between protagonist centrality metrics and movie ratings (Table 3), only relative betweenness centrality was found to be significantly positively correlated with movie ratings (*p* < 0.05). A positive correlation was also observed between the relative effective size of the protagonist and movie ratings.

### 3.3. Analysis of Protagonist Ego Networks

**Figure 6:**
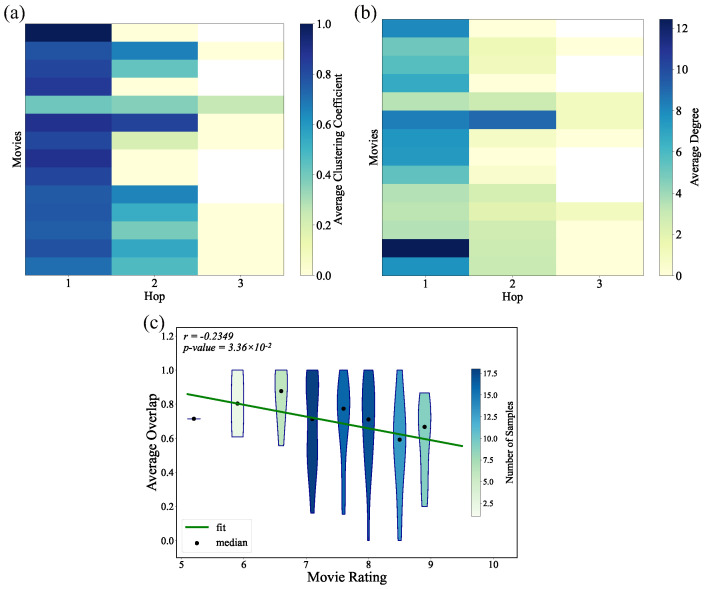
Heatmaps illustrating the relationships between movie ratings and the subnetworks constructed by unions of ego networks at different hops from different main characters: (**a**) average clustering coefficient and (**b**) average degree. (**c**) Correlation between the relative node overlap ratio and movie ratings.

The study revealed that 68 out of 82 networks (82.9%) could be fully encompassed within the one-hop union off their respective main character ego networks. As shown in Figure 6, for the remaining 14 films, networks formed by the union of the one-hop ego networks of all main characters exhibited significantly higher average clustering coefficients and average degrees than those of size-matched random networks, as confirmed through Z-score comparisons. Additionally, a statistically significant negative correlation was found between movie ratings and the relative node overlap proportion of main character ego networks (the ratio of the number of nodes in the intersection to the number of nodes in the union of their node sets). This suggests that higher audience ratings are associated with lower overlap among protagonists’ ego networks.

### 3.4. Analysis of Network Connectivity

**Figure 7:**
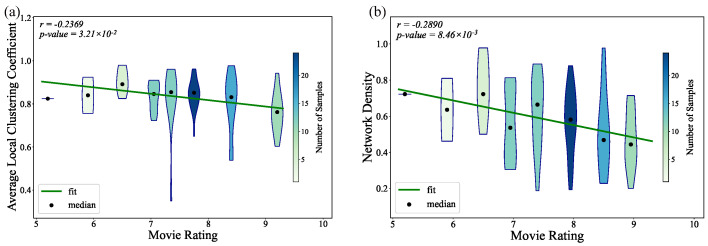
Relationship between movie ratings and (**a**) the average local clustering coefficient of movie character networks and (**b**) the network density of movie character networks.

As shown in Figure 7, there is a negative correlation between both network density and the average local clustering coefficient and movie ratings. Higher network density, which reflects frequent direct interactions between characters, is associated with lower ratings. This suggests that films with more densely connected character networks tend to receive lower ratings from audiences.

### 3.5. Analysis of Network Information Transmission Efficiency

**Figure 8:**
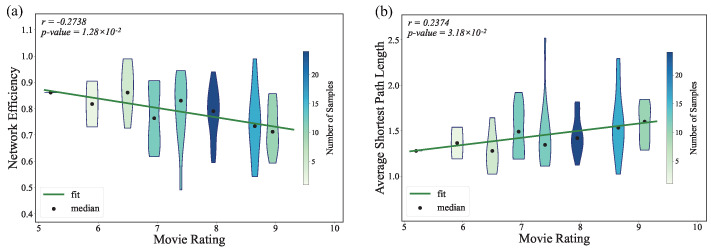
The violin plots showing the relationship between movie ratings and (**a**) the network efficiency of movie character networks and (**b**) the ASPL of movie character networks.

As shown in Figure 8, the ASPL is positively correlated with movie ratings. The shortest path captures the cost each node pays to obtain information, and a longer path can conceal certain details. When the distance is calibrated well, such as requiring the protagonist to reason before uncovering key facts, it introduces suspense into the plot. A negative correlation between network efficiency and ratings supports the same idea. Building more indirect links therefore showcases the author’s narrative skill more effectively than detailing every direct interaction between the protagonist and other characters.

### 3.6. Analysis of the Hierarchy of Character Network Structure

**Figure 9:**
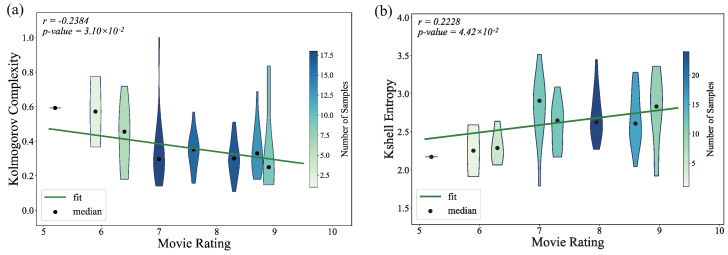
Relationship between movie ratings and (**a**) the Kolmogorov complexity of movie character networks and (**b**) k-shell entropy.

As shown in Figure 9, a negative correlation was observed between movie ratings and network density, whereas ratings were positively correlated with the ASPL. These patterns suggest that networks in higher-rated films may exhibit stronger hierarchical organization. To examine this possibility, we analyzed two additional complexity measures, namely k-shell entropy and Kolmogorov complexity. The results show a positive correlation between k-shell entropy and ratings and a negative correlation between Kolmogorov complexity, approximated by the gzip compression ratio of the adjacency matrix [31] and ratings.

### 3.7. Analysis of the Types of Character Relationships

**Figure 10:**
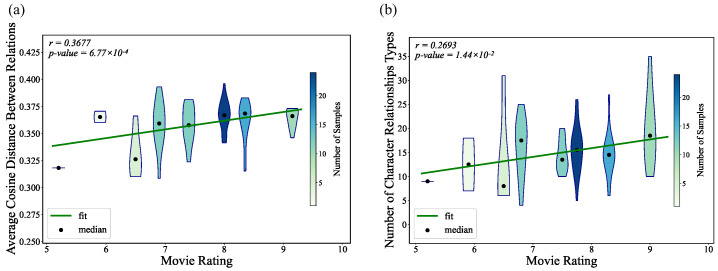
Relationship between movie ratings and (**a**) the number of edge relationship types in the character network and (**b**) the average cosine distance among relation word vectors for each movie following the conversion of edge relationships into word vectors.

As shown in Figure 10, the number of character-relationship types in a film is significantly and positively correlated with the film’s rating. After mapping each relationship to a sentence-level semantic embedding with the gte-multilingual-base model, we also find a significant positive correlation between the film’s rating and the average cosine distance among the relationship vectors; the same result emerges when we repeat the analysis with the ernie-1.0-base-zh and chinese-roberta-wwm-ext models. Semantic similarity is a common task in natural language processing, and the models we selected perform exceptionally well in Chinese semantic discrimination tasks. This allows us to transform the relationships in the network into vectors, accurately capturing the complex emotions and relational semantics in animated films.

## 4. Discussion

As shown in Table 1, and consistent with Naoki Masuda’s observations on comic book character networks [28], only four films in our sample display positive degree assortativity coefficients. This outcome differs from the pattern commonly seen in social networks [29], yet agrees with earlier findings for ego networks [30]. The prevailing negative assortativity indicates asymmetric character interactions. Unlike real social structures such as the Zachary Karate Club network or standard ego networks, movie character networks typically revolve around one or more central protagonists, creating configurations that are explicitly goal oriented and plot driven. Compared with general social networks [32,33,34,35,36], they tend to have shorter average path lengths and higher densities, features that are intentionally crafted to accelerate storytelling and enhance narrative efficiency. Further analysis of protagonist-centered ego networks supports this view. In most films, the entire character network falls within a protagonist’s one hop neighborhood; even when full coverage is not achieved, the subnetwork inside that neighborhood exhibits significantly higher mean degree and clustering coefficients, with Z scores well above those of random networks. Higher film ratings are also associated with lower overlap among the ego networks of multiple protagonists, suggesting that successful movies create relatively independent social spaces for each lead character and thus accommodate multithreaded and multi-perspective narratives. Compared with single-protagonist linear stories, this distributed center arrangement allows richer plot development and stronger character tension.

From a network-metric perspective, highly rated films present character networks with relatively low density and clustering coefficients, giving rise to a structure that is loosely connected yet orderly. This looseness offers greater narrative flexibility by reducing redundant plotlines that often emerge when inter-character links are too tight. To preserve narrative coherence, however, such networks still sustain shorter average path lengths and slightly higher densities than those found in most real-world social networks, making them compact without becoming overly intricate. Within our dataset, the higher-rated titles also display somewhat longer ASPL and lower information-transmission efficiency, features that provide room for storytelling devices such as information delays, misunderstandings, and plot twists, thereby intensifying suspense and pacing. A k-shell decomposition further reveals strong hierarchical organization with multi-layered relationships that let characters influence one another both within and across levels and enrich emotional conflict and plot development. In addition, the negative correlation between Kolmogorov complexity and film ratings shows that successful films preserve regular connection patterns while retaining diverse hierarchical layers. This balance of order and variety adds depth and texture without overwhelming viewers, and it sustains emotional tension while keeping the storyline clear.

At the node level, studies have shown that variation in character centrality values, including degree, betweenness, and harmonic centrality, is significantly and positively correlated with movie ratings. These differences in centrality reflect variations in each character’s narrative importance or influence, which in turn encourage more complex interactions and enrich the story. Further analysis indicates that most of this variation arises from the gap between protagonists and supporting characters. As demonstrated in Table 3, the protagonist’s relative betweenness centrality is strongly positively correlated with ratings. Betweenness centrality measures a character’s capacity to serve as a bridge among otherwise separate nodes in the network; a protagonist who scores highly in this metric links disparate plotlines and character groups, thereby advancing the story and weaving different strands together. In *Zootopia*, for example, Nick Wilde, though not directly connected to many characters, acts as the key link among communities such as the city government, the First Precinct, and the Glacier Town mafia. The positive correlation between the protagonist’s effective size and film ratings provides additional support for this conclusion.

In addition to topology, the semantics of character relationships also shape film quality. Films with higher ratings typically display both a wider variety of relationship types and greater semantic dispersion. We measured this dispersion by embedding each relationship in a vector space and calculating the average cosine distance; larger values indicate more diverse relational meaning. Semantic diversity supports more complex and dynamic interaction patterns. For example, Castle in the Sky builds several relational configurations around broad themes such as environmentalism and war and reaches a relatively large cosine distance. If every character simply supported the protagonist, the story would become monotonous; if every character opposed the protagonist, plot development would stall. By combining support and conflict, intimacy and distance, the film creates multidimensional characters and leaves room for unpredictable plot development, which deepens the narrative and strengthens audience engagement.

The relationship between network metrics and narrative theory is closely intertwined. Character networks reflect interactions that drive plot development. Narrative complexity is linked to several network metrics, such as character centrality, relationship semantics, network density, k-core layers, and Kolmogorov complexity. However, increasing narrative complexity does not monotonically affect movie ratings. Our analysis shows that a prominent protagonist, centrality differences among characters, more layered structures, and richer relationship variety contribute to narrative depth. Multiple storylines can be analyzed through the overlap of characters connected to the protagonist. Films with multiple protagonists or distinct communication ranges between them are often preferred by audiences. In scriptwriting, character relationships evolve as heterogeneous agents on a network, and the unpredictability of this process enhances the film’s narrative potential.

In summary, structural features such as low density, low clustering coefficients, and relatively long ASPL in character networks collectively produce a relational system that is loosely connected yet hierarchically organized. Several highly central characters together create a distributed centrality pattern that greatly increases the potential for parallel narrative threads to advance. Furthermore, the relatively small overlap of ego networks and the functional differentiation among protagonists preserve tension between characters while also adding to the overall structural complexity of the network. From the semantic perspective, a wide range of relationship types fosters multiple character groups in dynamic equilibrium, thereby strengthening the multidimensional drivers of plot development. Taken together, this character network architecture improves the scalability of the narrative system and furnishes a strong structural foundation for multithreaded storytelling, plot reversals, and emotional fluctuations, ultimately enabling a high degree of narrative flexibility and expressive potential.

## 5. Conclusions

In this paper, we integrate narrative theory with network science to quantify how character-interaction topology relates to audience ratings in 82 animated films. Screenplay-level interactions were transformed into character networks and evaluated across seven dimensions: influence disparity among characters; sources of influence disparity; overlap among main characters’ spheres of influence; information-transmission efficiency; network density and clustering coefficient; network hierarchy and regularity; and semantic diversity of relationship types.

Our analysis uncovers a consistent structural signature in higher-rated films. First, sizable gaps in centrality between protagonists and supporting characters point to a distributed-center arrangement that facilitates multiple interwoven plotlines. Second, networks with lower density and clustering remain navigable, allowing suspense, information delays, and plot twists while preserving coherence. Third, broader and more dispersed relationship semantics enrich thematic variety and emotional depth. Taken together, these traits indicate that loosely coupled, hierarchically layered, and semantically varied character networks enhance narrative flexibility and audience engagement.

These findings provide a data-driven toolkit for storytellers, offering practical guidance for early script development and translating readily to other narrative forms such as novels, television series, and comics.

## Figures and Tables

**Table 1:** Basic network properties of 82 animated films.

	Number of Nodes	Number of Edges	Average Degree	Degree Assortativity Coefficient	Network Density	Average Shortest Path Length
Mean	16.40	71.01	8.02	−0.28	0.57	1.48
Std	6.80	50.20	3.24	0.15	0.20	0.28
Min	6.00	8.00	2.67	−0.60	0.19	1.02
Max	40.00	210.00	17.48	0.14	0.98	2.52

**Table 2:** Correlation analysis between the CV of centrality measures and relative centrality measures of the protagonist.

Centrality Measures	Degree	Harmonic	Betweenness
Pearson correlation coefficient	0.8091	0.6073	0.7206
*p*-value	$3.68\times 10^{-20}$	$1.46\times 10^{-9}$	$2.31\times 10^{-14}$

**Table 3:** Correlation analysis between the protagonist’s relative centrality measures and movie ratings.

Centrality Measures	Degree	Harmonic	Betweenness
Pearson correlation coefficient	0.21	0.21	0.23
*p*-value	0.054	0.063	0.035

## Data Availability

The datasets generated and analyzed during the current study are available at Supplementary Materials.

## Supplementary Materials

The following supporting information can be downloaded at https://www.mdpi.com/article/10.3390/e27090914/s1.
